# Breast ultrasound: recommendations for information to women and referring physicians by the European Society of Breast Imaging

**DOI:** 10.1007/s13244-018-0636-z

**Published:** 2018-08-09

**Authors:** Andrew Evans, Rubina M. Trimboli, Alexandra Athanasiou, Corinne Balleyguier, Pascal A. Baltzer, Ulrich Bick, Julia Camps Herrero, Paola Clauser, Catherine Colin, Eleanor Cornford, Eva M. Fallenberg, Michael H. Fuchsjaeger, Fiona J. Gilbert, Thomas H. Helbich, Karen Kinkel, Sylvia H. Heywang-Köbrunner, Christiane K. Kuhl, Ritse M. Mann, Laura Martincich, Pietro Panizza, Federica Pediconi, Ruud M. Pijnappel, Katja Pinker, Sophia Zackrisson, Gabor Forrai, Francesco Sardanelli, for the European Society of Breast Imaging (EUSOBI) , with language review by Europa Donna–The European Breast Cancer Coalition

**Affiliations:** 1 Dundee Cancer Centre, Clinical Research Centre, Ninewells Hospital and Medical School, Tom McDonald Avenue, Dundee, UK; 2 PhD Course in Integrative Biomedical Research, Department of Biomedical Science for Health, Università degli Studi di Milano, Via Mangiagalli, 31, 20133 Milan, Italy; 3 Breast Imaging Department, MITERA Hospital, 6, Erithrou Stavrou Str. 151 23 Marousi, Athens, Greece; 4 Department of Radiology, Gustave-Roussy Cancer Campus, 114 Rue Edouard Vaillant, 94800 Villejuif, France; 5 Department of Biomedical Imaging and Image-guided Therapy, Division of Molecular and Gender Imaging, Medical University of Vienna, Währinger Gürtel 18-20, 1090 Wien, Austria; 6 Clinic of Radiology, Charité Universitätsmedizin Berlin, 10117 Berlin, Germany; 7 Department of Radiology, University Hospital of La Ribera, Carretera de Corbera, Km 1, 46600 Alzira, Valencia Spain; 8 Radiology Unit, Hospices Civils de Lyon, Centre Hospitalo-Universitaire Femme Mère Enfant, 59 Boulevard Pinel, 69 677 Bron Cedex, France; 9 Thirlestaine Breast Centre, Cheltenham General Hospital, Thirlestaine Road, Cheltenham, GL53 7AP UK; 10 Division of General Radiology, Department of Radiology, Medical University Graz, Auenbruggerplatz 9, 8036 Graz, Austria; 11 Department of Radiology, University of Cambridge, Cambridge Biomedical Campus, Hills road, Cambridge, CB2 0QQ UK; 12 Institut de Radiologie, Clinique des Grangettes, Chemin des Grangettes 7, 1224 Chêne-Bougeries, Genève Switzerland; 13 Referenzzentrum Mammographie München and FFB gGmbH München, Sonnenstraße 29, 80331 Munich, Germany; 14 University Hospital of Aachen, Rheinisch-Westfälische Technische Hochschule, Pauwelsstraße 30, 52074 Aachen, Germany; 15 Department of Radiology, Radboud University Nijmegen Medical Centre, Geert Grooteplein Zuid 10, 6525 GA Nijmegen, The Netherlands; 16 Unità Operativa Radiodiagnostica, Candiolo Cancer Institute - FPO, IRCCS, Str. Prov. 142, km 3.95, 10060 Candiolo, Turin Italy; 17 Breast Imaging Unit, Scientific Institute (IRCCS) Ospedale San Raffaele, Via Olgettina, 60, 20132 Milan, Italy; 18 Department of Radiological, Oncological and Pathological Sciences, Sapienza University, Viale Regina Elena, 324, 00161 Rome, Italy; 19 Department of Imaging, University Medical Centre Utrecht, Heidelberglaan 100, 3584 CX Utrecht, The Netherlands; 20 Department of Radiology, Breast Imaging Service, Memorial Sloan Kettering Cancer Center, 300 E 66th Street, New York, NY 10065 USA; 21 Diagnostic Radiology, Department of Translational Medicine, Faculty of Medicine, Lund University, Skåne University Hospital Malmö, SE-205 02 Malmö, Sweden; 22 Department of Radiology, Duna Medical Center, Budapest, Hungary; 23 Department of Biomedical Sciences for Health, Università degli Studi di Milano, Via Morandi 30, 20097 San Donato Milanese, Milan Italy; 24 Unit of Radiology, IRCCS Policlinico San Donato, Via Morandi 30, 20097 San Donato Milanese, Milan Italy

**Keywords:** Breast cancer, Breast ultrasound (US), BI-RADS, Colour-Doppler, Elastography, Automated whole breast ultrasound

## Abstract

**Abstract:**

This article summarises the information that should be provided to women and referring physicians about breast ultrasound (US). After explaining the physical principles, technical procedure and safety of US, information is given about its ability to make a correct diagnosis, depending on the setting in which it is applied. The following *definite indications* for breast US in female subjects are proposed: palpable lump; axillary adenopathy; first diagnostic approach for clinical abnormalities under 40 and in pregnant or lactating women; suspicious abnormalities at mammography or magnetic resonance imaging (MRI); suspicious nipple discharge; recent nipple inversion; skin retraction; breast inflammation; abnormalities in the area of the surgical scar after breast conserving surgery or mastectomy; abnormalities in the presence of breast implants; screening high-risk women, especially when MRI is not performed; loco-regional staging of a known breast cancer, when MRI is not performed; guidance for percutaneous interventions (needle biopsy, pre-surgical localisation, fluid collection drainage); monitoring patients with breast cancer receiving neo-adjuvant therapy, when MRI is not performed. *Possible indications* such as supplemental screening after mammography for women aged 40–74 with dense breasts are also listed. Moreover, *inappropriate indications* include screening for breast cancer as a stand-alone alternative to mammography. The structure and organisation of the breast US report and of classification systems such as the BI-RADS and consequent management recommendations are illustrated. Information about additional or new US technologies (colour-Doppler, elastography, and automated whole breast US) is also provided. Finally, five frequently asked questions are answered.

**Teaching Points:**

*• US is an established tool for suspected cancers at all ages and also the method of choice under 40.*

*• For US-visible suspicious lesions, US-guided biopsy is preferred, even for palpable findings.*

*• High-risk women can be screened with US, especially when MRI cannot be performed.*

*• Supplemental US increases cancer detection but also false positives, biopsy rate and follow-up exams.*

*• Breast US is inappropriate as a stand-alone screening method.*

## Introduction

Breast ultrasound (US) is one of the four main methods for diagnosing breast diseases, together with mammography, magnetic resonance imaging (MRI), and image-guided needle biopsy.

This article is the third in a series of recommendations for information for women issued by the European Society of Breast Imaging (EUSOBI), the first focusing on mammography [1, 2] and the second on breast MRI [3]. It is addressed to the women themselves and to physicians dealing with patients for whom breast US is, or may be, under consideration. In particular, seven special information notes (from A to G) and five frequently asked questions are formulated for use in direct communication with women. Considering the relevant differences across European countries in terms of available technology, national guidelines, clinical practices, health care systems, and insurance coverage, these recommendations can have different applications under local conditions.

A search on the PubMed/Medline has been performed for papers published from January 2007 to December 2016, subsequently updated to December 2017, using the terms “breast” AND “ultrasound”. Articles with an informative content most suitable for the aim of the article were selected as references with special regard to predetermined issues: safety/quality; protocols and techniques; test performance (sensitivity and specificity); screening and clinical indications. Other articles were added as references as suggested by the authors.

The entire text underwent a double evaluation by the authors, each of them contributing with relevant intellectual content. However, as many different issues are considered, single authors generally agreeing on these recommendations may have different opinions on individual statements.

## General issues, safety, and quality

Ultrasound is an imaging method based on the application of sound waves, i.e. not radiation but mechanical periodic compression/rarefaction waves of a medium, for producing images of the internal body structures. In particular, this method uses sound waves with frequencies above the upper limit of human hearing (limited from 16 Hz to 20 kHz), indeed defined as *ultrasound*, in particular frequencies of at least 1 MHz, able to penetrate biologic tissues. Images are obtained by sending pulses of US waves into the tissues using a probe, which is a transducer that can transmit and receive US. Gel, which is used as a propagation medium, is interposed between the US probe and the skin of the region to be examined. These waves are variably reflected as echoes by the tissues, from which comes the denomination of *echography*, other equivalent denominations being *sonography* or *ultrasonography*. Reflected echoes are received by the probe and forwarded as electronic signals to a computer system that finally generates the images [4, 5].

Importantly, US is a technique that does not expose the woman to radiation and its related risks. The US waves used for medical imaging do not cause injury to human tissues, allowing a very safe diagnostic approach [4–6]. There are no contraindications to breast US. Practical difficulties can be encountered in women with severe disabilities preventing the correct positioning or in obese women due to limited penetration depth. The cost of breast US is comparable to that of mammography and much lower than that of breast MRI, although the time spent by the physician performing the examination is usually not (or only scarcely) included in the reimbursement evaluation.

Assurance of the technical quality of US equipment should follow specific protocols [7, 8]. As a general suggestion, women should be aware that US equipment older than 10 years may not yield state-of-the-art examination results [8, 9].

When using the most common modality of US image generation (i.e. with the probe manually moved on the body surface of a patient — *handheld,* also called *manual*, US), the diagnostic performance of breast US is dramatically influenced by the specific competence and experience of the operator. As a consequence, *handheld US of the breast should only be carried out by specially trained and experienced physicians, preferentially a breast radiologist.* In fact, in the vast majority of European countries, *handheld* breast US is performed by physicians, not by radiographers or so-called “sonographers”. Of note, although automated three-dimensional whole-breast US systems can be used by radiographers for generating three-dimensional US datasets [10], the interpretation of the images always requires the experience of an expert in handheld breast US to keep false positive and false negative calls as low as possible [11]. In the case of suspicious findings with automated breast US, handheld US should be performed to confirm the finding and make a clinical decision [12, 13].

During handheld breast US, the physician chooses images to capture and makes them available with the report. In fact, unlike other breast imaging methods such as mammography or MRI, the vast majority of the handheld US examination is not recorded. Of note, many experienced breast radiologists use breast US for targeted investigations of areas of concern, documenting abnormalities that she/he has noticed during the scanning process. When a whole breast handheld examination is performed, at least one image of normal findings for each quadrant and for the retroareolar region, optionally for the axilla, should be documented. Notably, if an examiner overlooks an abnormality during the US examination, that abnormality will not be available for later reviews of that US examination. This, in turn, means that quality assurance for handheld breast US is more challenging than it is for other breast imaging methods, due to a higher proportion of human variability in determining the diagnostic result: *handheld breast US is highly operator-dependent*. Third parties cannot review a previous study with the only exception of those findings identified and documented with images by the original examiner. However, this is not true for *automated whole breast US*, a standardised approach, where the whole examination is recorded, even though this technique suffers from other specific limitations (see below).

A *breast radiologist* can compare US findings with those from mammography and MRI, integrate clinical information, appropriately define further work-up in terms of time-interval and imaging modalities to be used and, when indicated, perform an imaging-guided percutaneous biopsy. *EUSOBI strongly encourages women to ask for a dedicated breast radiologist to perform their breast US*.

- ***Note A.*** *The high operator-dependence of hand-held breast US implies that the specific experience and expertise of the examiner strongly determines the reliability of the results. This means that breast radiologists are the most suitable professionals for performing handheld breast US. In fact, they are able to check for appropriateness of the breast US examination, to provide correlation with other breast imaging studies already performed (mammography or MRI), to propose and perform further imaging work-up or needle biopsy, and to define the correct interval time to the next follow-up breast imaging study. Breast US is one of the cases where the expertise of the examiner is of greater importance than the quality of the technical equipment. Notably, guidelines suggest, as a general rule, that equipment older than 10 years should be replaced* [9]*. Thus, ask for a breast radiologist to perform your breast US and ask for dedicated, not outdated, US equipment.*

## Examination protocol and technical issues

Clear instructions and explanations regarding the entire procedure should be provided to the woman. The optimal protocol for performing breast US requires the patient to lie in the supine position with the chest undressed and with arms flexed behind the head to flatten the breast or in the oblique position for scanning the lateral part of the breast and, when needed, axilla [13]. Breast US can even be performed in women who cannot lie down or flex the arms behind the head, although in these conditions it becomes more difficult to perform the examination and the results might be suboptimal.

A clinical US scanner with dedicated linear probes working from 10 to 15–18 MHz for the superficial details or small breasts, and from 4 to 8 MHz for deeper areas or very large breasts, should be employed. A contact gel is always applied to the breast to allow the US waves to be sent from the probe to the breast tissues. The probe is placed on the breast and moved in multiple directions, with a gentle pressure that makes US usually painless and well tolerated.

Importantly, for many indications, breast US is aimed at evaluating specific clinical findings or findings detected through other imaging modalities. In many cases, only a unilateral, targeted breast US examination is performed and not a bilateral whole breast US. Thus, whether both breasts, only one breast or just a part of one breast will be scanned depends on the indication, mammographic breast density, and local policies. The axilla will be scanned in women with clinical or radiological abnormalities, in women with personal history of breast cancer, and whenever the radiologist evaluates this to be useful. Of note, routine axillary scanning during screening breast US after negative mammography showed no effect on additional cancer detection but increased the number of false-positive results [14]. If suspicious nodes are seen in the axilla, other nearby lymph node stations (i.e. supra- or infra-clavicular locations, in the neck or below the clavicle) may be examined.

- ***Note B.*** *Follow carefully the instructions of the radiologists during the breast US examination. She/he will ask you to move your trunk or your arms to get the best position for scanning each of your breasts. Your cooperation is needed to get the best diagnostic performance and reduce the examination time. A certain pressure of the probe on your breast is necessary to avoid artefacts,* *i.e.* *unwanted alterations of the images possibly reducing the diagnostic power of the examination. If the pressure is uncomfortable, please tell the examiner immediately.*

### Additional US techniques

Both *colour-Doppler* and *elastography* are special tools that may be available on US machines. Nowadays, almost all US equipment provide colour-Doppler images. Elastography is much less widespread and currently used only in some centers for particular cases.

*Colour-Doppler* exploits the Doppler effect, a physical phenomenon according to which the frequency or wavelength of a wave changes for an observer who is moving relative to the wave source or vice versa. A common example is the change of pitch heard when a vehicle sounding a siren approaches, passes, and recedes. Colour-Doppler representation of the vessels, obtained using no or minimal pressure to prevent vessels from collapsing [15], are usually superimposed on standard grey-scale US images (*duplex* modality). This approach allows for identifying vessels in the context of breast tissue, in particular around and inside mass lesions. The presence of vessels can be an additional criterion aiding the differentiation of malignant from benign lesions, but is not sufficient alone to characterise a lesion [13, 16]. Moreover, when performing a US-guided biopsy, colour-Doppler can be useful to visualise the course of the vessels in order to avoid crossing them with the needle, causing haematoma [17].

*Elastography* measures tissue stiffness looking at modifications of the US image of a given lesion after applying a mechanical stress [18]. The stress is mostly applied as a manual pressure, requiring a very gentle quavering of the probe (not causing any discomfort) or using ultrasound waves as a source of mechanical stress (*shear wave elastography*) [19]. The premise for interpreting the results of this tool is that malignant tissues are mostly, but not always stiffer than benign tissues. Colour-coded maps are provided for visual representation of the results. When using the shear wave technique, a lesion stiffness quantification is given (in kilopascal, kPa, or in m/s). Using this approach, a significant increase in specificity has been reported [20]. Elastography evaluation of breast lesions has been recently included as an associated feature in the BI-RADS system [21] (see below for BI-RADS categories).

Both colour-Doppler and elastography are, however, not mandatory elements of a breast US examination. Other complementary ultrasound techniques, such as *harmonic imaging* and *compound US imaging* may be associated to grey-scale images.

### Automated whole breast US

Automated whole breast US, approved by the Food and Drug Administration in 2009, offers the potential for acquiring a volumetric three-dimensional breast dataset with a standardise examination protocol [22, 23]. It can be performed by a radiographer/technician, with a total time for patient preparation and acquisition ranging approximately between 10 and 15 min [10]. A longer time is needed when multiple acquisitions have to be performed in large breasts. As explained above, automated image acquisition still implies interpretation and reporting by an expert in handheld breast US at a later time. If findings warrant further assessment, the woman needs to be recalled for a handheld US. The main advantage of automated breast US is a reduced operator dependence, resulting in a higher reproducibility of the examination. The main disadvantages are the need of recall in the case of positive examination a number of additional false positive findings, with an increase of the recall rate of 28.5 per 100 screened women [23]. The possibility of using this technique as an additional tool for screening women with dense breasts without other risk factors has been explored, with a cancer detection rate additional to mammography up to 2.4 per 1000 women screened [24]. More well-designed large studies are welcome.

As automated breast US is still not widely used, in this article when the term *breast US* is used without specifications, we mean *handheld breast US*.

- ***Note C.*** *The experience with automated breast US is promising but still relatively limited. If the woman is examined with this technique, it’s important to remember that the report is the result of an evaluation performed by a radiologist in a separate session and that, if a suspicious finding is detected, the woman has to be recalled and a work-up with targeted handheld US is necessary.*

### After the procedure

When the examination is completed, the woman is provided with a paper or tissue towel for removing the residual gel. She can get dressed again and goes home. The report is usually generated at the end of the examination. When correlation with a complex clinical history and/or other imaging modalities is needed, the report may need more time to be generated. In the majority of cases, the breast radiologist will talk directly to the woman immediately after the end of the examination. The time for report availability can vary across countries and centres. For the case of automated breast US, see above the Note C.

## Test performance

Performance refers to the general ability of a test, here breast US, to make a correct diagnosis, i.e. to see cancers when they are present (sensitivity) or to exclude them when they are not (specificity).

No test is perfect. This is also true for breast US [6, 25–27]. Its sensitivity and specificity also depend on the specific setting (i.e. the indication) in which it is applied, particularly in the major distinction between its application in symptomatic women (*diagnostic performance*) or in asymptomatic women (*screening performance*).

Considering the high operator-dependence, and the fact that most US studies are performed together with, and complementary to, other examinations (clinical examination and mammography), it is difficult to define precisely a sensitivity for US alone. It varies strongly depending on lesion size, type of breast tissue and (as for all methods) on patient selection.

When a woman has focal symptoms, typically a palpable lump, US is performed as a targeted examination and has a high sensitivity [6]. In this clinical setting, US is useful for differentiating liquid benign lesions (cysts) from solid masses, characterising solid masses, and deciding whether a US-guided needle biopsy should be performed [21]. Since the negative predictive value (NPV) of breast US, i.e. the probability that a negative examination is truly negative (no cancers are present) is usually not perfect (like that of mammography), exclusion of malignancy can require a combined evaluation of ultrasound with mammography and clinical findings. Based on US, it is possible to exclude malignancy in case of pathognomonic findings like simple cysts or when a low suspicion finding (like some mammographic asymmetry) correlates at US with homogeneously hyperechoic normal gland tissue or with a mobile and elastic well-circumscribed oval mass compatible with a fibroadenoma. The addition of mammography to US should be considered whenever the pre-test probability of malignancy is high enough, usually in women over 40 or in women with somewhat atypical US findings.

Sensitivity of breast US is high for characterising mammographic masses, lower in in the presence of isolated calcifications, i.e. of those without an associated mass. If the suspicion of cancer persists after targeted US or the lesion cannot be clearly identified at US, further work-up is necessary such as a needle biopsy under mammographic guidance or, in particular cases, contrast-enhanced MRI or contrast-enhanced mammography.

In women with dense breasts, US screening is able to detect additional cancers, described to be from 2 to 7 every 1000 negative mammograms [27, 28], to be considered as the combined result of the limitations of mammography in the presence of dense breasts and of other associated risk factors. High rates (over 2 per 1000 mammograms) of additional cancer detection rate may be due to population preselection. The masking effect of breast density appears to be lower for US than for mammography. In general, the sensitivity of US is good, even for small cancers that occur in dense (usually hyperechoic) surrounding tissue and present as masses, with or without microcalcifications. The sensitivity decreases for masses in large breasts, in fatty breasts, and in breasts with inhomogeneous breast tissue, either by many interposed fatty components or due to mastopathy changes, being strongly variable with lesion size, palpability, composition of the surrounding tissue, and breast size.

Notably, breast US also detects a variety of non-palpable benign lesions, which are very common in the breast but may otherwise have gone unnoticed, sometimes requiring needle biopsy, showing their benignancy (false positives). This is one of the major drawbacks of the use of breast US as a screening tool. A systematic review of supplemental screening in women with dense breasts reported very low positive predictive value of both handheld and automated breast US (from 3.2 to 7.5%) and a biopsy rate as high as about 6% [29].

Invasive cancers not visible or not detected on US are in general either very small, behind the nipple or lesions which are difficult to distinguish from normal gland or fat tissue or from fibrocystic changes, such as invasive lobular cancers, accounting for up to 15% of all breast cancers [30]. US may also miss microinvasive cancers and diffusely growing cancers. Accordingly, suspicious findings at clinical examination, mammography, or MRI should not be dismissed because of a normal US, even when US has been targeted to the region thought to harbour the finding. Moreover, US is less sensitive than mammography for non-invasive breast cancers (ductal carcinoma in situ, DCIS) commonly detected at mammography due to the presence of calcifications [31].

- ***Note D.*** *When both mammography and breast US are requested, US should be performed after mammography. The best scenario is that both mammography and US are evaluated at the same time from the same radiologist providing a unique conclusion from the two examinations. Your radiologist may recommend an adjunct US if you have very dense breasts and a negative mammogram, also taking into account other risk factors. If you feel a palpable lump, US may be the first-line examination used, especially before 40 years of age.*

## Indications for breast US

Definite, possible, and inappropriate indications for breast US are listed in Tables 1, 2, and 3, respectively. In the following paragraphs we will enter into the details for those indications being most clinically relevant or debated.

**Table 1:** Definite indications for breast ultrasound

Palpable lump	
Axillary adenopathy	
First approach for clinical breast abnormalities under age 40	
First approach for clinical breast abnormalities in pregnant or lactating women	
Suspicious abnormalities at mammography or magnetic resonance imaging (MRI)	
Suspicious nipple discharge	
Recent nipple inversion	
Skin retraction	
Breast inflammation	
Abnormalities at the surgical scar after breast conserving surgery or mastectomy	
Abnormalities in the presence of breast implants	
Screening high-risk women, especially when MRI is not performed	
Loco-regional staging of a known breast cancer, when MRI is not performed	
Guidance for percutaneous breast interventions (needle biopsy, pre-surgical localisation, fluid collection drainage)	
Monitoring patients with breast cancer receiving neo-adjuvant therapy, when MRI is not performed)

**Table 2:** Possible indications for breast ultrasound

Supplemental screening after mammography for women aged 40–74 with dense breasts	
Surveillance of women with previous mammographically occult breast cancer	
Palpable lump felt by the woman with normal clinical examination	
Focal new breast pain unrelated to the menstrual cycle	
Intraoperative US lesion identification and US of specimens

**Table 3:** Inappropriate indications for breast ultrasound

Screening for breast cancer in average-risk women under age 40	
Screening for breast cancer as a stand-alone alternative to mammography in average-risk women with ≥40 years of age	
Follow-up in women with previous history of breast cancer as an alternative to mammography	
Diffuse bilateral premenstrual breast pain	
Screening of breast implant integrity in asymptomatic women	

### Screening

In high-risk women who cannot undergo screening with MRI, US is indicated in very young women under about 30–35 years of age and as an adjunct to mammography in older women [32, 33]. Of note, in BRCA mutation carriers, evidence of higher sensitivity to radiation exposure of the breast tissue [34] suggests avoiding mammography, at least at young age.

In women at average or intermediate risk with dense breasts, supplemental screening with either handheld or automated bilateral breast US can be performed after a negative mammogram to increase cancer detection, albeit at the price of a high false positive recall rate. Additionally, invasive cancers detected by US, which tend to be small and node negative, are thus more likely to be treated with curative intent [35]. Taking into consideration that interval cancers are those diagnosed in the time interval between two scheduled screening examinations, the addition of US screening to mammography in women with dense breasts has been shown in one study [36] to reduce the interval cancer rate to a level similar to that observed in women with non-dense breasts screened with mammography only. These results support implementation of research in randomised controlled trials on additional US in women with increased breast tissue density. A systematic review published in 2009 [37] reported that supplemental breast US screening after negative mammography in women with American College of Radiology (ACR) density 2–4 allowed a cancer detection rate of 0.32% (mean size 9.9 mm, negative nodal status), most cancers being detected in women with ACR density 3 or 4, with biopsy rates from 2.3% to 4.7% and a PPV from 8.4% to 13.7%, about one third of the PPV of mammography.

A recent study showed that the ability of US to detect breast cancers is comparable to that of mammography in asymptomatic women with heterogeneously or extremely dense breast tissue in at least one quadrant and at least one other risk factor for breast cancer, even though with a trade-off in terms of a higher rate of false positive recall and biopsy [38]. Disease-free and overall survival after detection with screening US were reported to be comparable with those after mammography in Chinese women [39]. However, *the performance of breast US in a population with a high prevalence of women with small and dense breasts cannot be translated to the European or American population.* In summary, adjunctive US screening can detect biologically important cancers, *but there is not sufficient evidence in favour of adopting adjunctive US screening as a general policy* [40]. *Well-designed large studies with sufficient follow-up are needed*.

When considering US screening, one relevant issue is the risk of false positive findings. Only fewer than one of ten biopsies prompted by US turns out to be malignant [41]. The high rate of biopsy poses major problems when applying this method to a population every one or two years: every woman might undergo one or two biopsies during her lifetime. Moreover, US screening is more time consuming than mammography for both the patient and the radiologist. Whereas breast US screening takes about 20 min to perform, the acquisition of a screening mammogram by a radiographer is completed in 5 min and it takes approximately 1–2 min to be interpreted by the radiologist [42]. Automated whole breast US allows the acquisition of imaging data of both breasts in about 15–20 min by a technician and must be read by a radiologist at a later time in about 5–9 min [11]. This approach implies a relatively high false positive rate [23, 43]. *Research to define the role of automated breast US is still needed.*

US screening instead of screening mammography is not recommended in women at average risk of breast cancer due to the combination of a possible reduced sensitivity (especially in fatty breasts) and a possible increase of false negatives, i.e. cancers missed.

### Diagnostic assessment

In patients with symptoms or signs suspicious for breast cancer, which typically comprise palpable lumps (but also, nipple inversion, localised skin retraction or other modifications, serous or bloody nipple discharge), US is an established tool complementing clinical examination and mammography at all ages. It is the method of choice for assessing breast lumps in young women. US can also reveal the origin of the symptoms in patients with painful cysts [44]. When patients present with unilateral, localised, non-cyclic breast pain, the chance of malignancy is as low as 1% [45]. In pregnant or lactating women with a palpable lump, focal breast pain, or nipple discharge, US is also the initial modality of choice for identifying most benign and malignant masses [25]. However, when needed, using adequate shielding, mammography can be supplemented even during pregnancy and injury to the unborn baby is not expected beyond week 16.

Patients presenting with diffuse breast pain, usually in association with the premenstrual phase, are often evaluated and treated only on a clinical basis.

### Assessment of lesions detected by mammography

Targeted US plays a complementary role when a mass or an asymmetry is seen on a mammogram. It may help in differentiating cystic from solid lesions and excluding or identifying a solid mass underlying an asymmetry. In the presence of clustered calcifications, particularly clusters larger than 10 mm, US can depict associated masses, ductal change, or intracystic lesions, cysts and microcysts that may be crucial for diagnosis [25, 46].

- ***Note E:*** *Mammography is clearly superior to US in identifying and characterising calcifications. However, in the presence of calcifications on mammography, targeted US is recommended to reveal possible underlying masses.*

### Assessment of lesions detected by MRI

Breast MRI is the most sensitive tool for diagnosing breast cancer [33], in particular when mammographic calcifications are absent [47, 48]. Of note, a variable number of lesions detected on MRI are not identifiable on mammography and US and performing an MRI-guided biopsy for each of these lesions may be disadvantageous, because most of them are benign. US plays an essential role in the work-up of MRI-detected lesions. In fact, even when an US performed before MRI did not reveal abnormalities, a *second-look targeted US after MRI may reveal the lesion*: from 46% to 71% of MRI-detected lesions can be identified by targeted US [25], more often for larger than for smaller lesions [49]. Identifying an MRI-detected lesion on US allows for planning a US-guided biopsy, which is more comfortable for the patient, cheaper, and less time consuming than an MRI-guided biopsy. Notably, to visualise MRI abnormalities on US, specific experience and skill are required, especially for the crucial issue of an accurate MRI-US spatial correlation. Differences in patient positioning during the two examinations may lead to difficulties in correlation and to incorrect false negative biopsies. Conversely, a lesion not identified with a pre-MRI US and identified with a post-MRI (second look) US does not mean that the first examination was not correctly performed: small lesions can go unnoticed at US when their location is unknown before MRI but become visible when MRI indicates their exact localisation. Marker placement and follow-up imaging after US-guided biopsy (see below) should be performed [25, 50, 51]. *In the case of US-guided biopsy of an MRI-detected lesions giving a pathological result not fitting with the MRI finding, MRI could be repeated. If an MRI-detected suspicious lesion is not recognised on US, an MRI-guided biopsy is mandatory* [52]*.*

### US-guided percutaneous interventions

Most breast cancers are detected by screening mammography or because of clinical symptoms. The standard workflow to assess suspicious lesions is based on mammography, US, and – when necessary – image-guided needle biopsy. Criteria were developed for decision making about biopsy of US-detected masses [21, 53, 54]. When a suspicious lesion is identifiable on US, US is the preferred image guidance technique for percutaneous needle biopsy [55, 56]. In fact, the procedure is fast, has low costs, allows for the use of smaller needles, is only minimally uncomfortable for the patient and does not expose the patient to radiation. US-guided breast biopsies offer great advantages over mammography or MRI guidance methods for needle biopsy. US-guided interventions can be requested not only by radiologists but also by referring physicians to established histopathologic diagnosis and allow treatment planning. *Open surgical diagnostic biopsies are performed only in a few particular cases, either when image-guided biopsy is not possible or due to patient’s preference.*

Independent of whether a mass is palpable or not, US guidance allows the operator to follow the procedure in real time and to verify the needle position for improving the overall accuracy. The patient position during biopsy is quite similar to the US examination. Breast compression is not required. The skin is disinfected and local anaesthesia is injected. A small incision of the skin is sometimes made at the entry site for an easier needle passage. The current good practice is the use of a 14-gauge or larger needle (which means using needles identified with smaller gauge numbers) to obtain three to six samples [50, 51]. Markers visible at various imaging modalities may be positioned at the biopsy site under US guidance, especially when there is a chance of masking or the disappearance of the biopsied lesion due to bleeding or very small size. This final step allows the evaluation of the concordance of the biopsy position as compared with other imaging modalities and, in case of neo-adjuvant therapy (i.e. a systemic medical treatment adopted in the case of large tumours to be reduced before surgery), to have the tumour position marked also in the case of complete response to therapy.

US-guided needle biopsy is a minimally invasive and safe technique for obtaining histologic diagnosis of a breast mass. The risk of complications such as infections and large haematomas (sometimes with pseudoaneurysms) is very low, approximately one every 1000 procedures [50, 51]. Further, pre-surgical localisation of non-palpable lesions can be similarly easily performed under US guidance in order to indicate the site or the disease extent to the surgeon. Mammography- and MRI-guided procedures (both biopsies and localisations) are reserved for those lesions not visible on US, as those procedures are more uncomfortable and more time consuming than under US guidance.

*During US-guided interventions, usually a nurse or a technologist assists the radiologist. More detailed information will be provided in a specific article dedicated to image-guided breast interventions.*

### Loco-regional staging

In women with a suspected breast cancer, detected with US or with other modalities, the whole breast harbouring the suspicious lesion, the ipsilateral axilla, and the contralateral breast can be examined with US. In fact, US has proven to be very valuable for assessing the size of invasive cancers, detecting other cancer foci in the affected breast, and identifying cancers in the contralateral breast [57–59]. Occult ipsilateral or contralateral cancers are more frequently found with US if the known cancer is palpable or larger than 2 cm, especially in the context of dense breasts [58].

However, the most accurate method for detecting additional ipsilateral and contralateral cancers in women with a newly diagnosed breast cancer is breast MRI, even though its routine preoperative use is still controversial [3]. At any rate, the additional findings of MRI can be considered for changing therapy planning only if they are pathologically verified to be malignant through a percutaneous biopsy. If an additional lesion seen on MRI is also visible on targeted (so-called *second look*, as mentioned above) US, the biopsy should be performed under US guidance [52]. If an additional lesion cannot be identified at targeted second-look US, the radiologist, together with a multidisciplinary team, will decide the next step, such as an MRI-guided biopsy or follow-up [3].

When an invasive cancer is newly diagnosed, the status of axillary lymph nodes is important for treatment planning, in particular for decision making about axillary treatment (i.e., surgical removal or radiation therapy of axillary lymph-nodes), and prognosis. Lymph nodes suspected of harbouring metastases can be revealed by US also in the absence of palpable axillary findings. In that case, US-guided percutaneous sampling concludes the diagnostic work-up, using fine needle aspiration or larger core needles [60].

### Evaluation of the effect of neoadjuvant therapy

Locally advanced cancers are defined as tumours that are larger than 5 cm in size in the absence of metastases to other organs or tumours of any size with direct extension to the chest wall or to the skin as well as with clinically fixed or matted axillary lymph-nodes, or any of infra-clavicular, supraclavicular, or internal mammary lymph-node involvement regardless of tumour stage [61]. These tumours are often treated with systemic treatment using different drugs before surgery, an approach referred as *neo-adjuvant therapy*. The aim is to reduce the tumour bulk, allowing for conservative surgery and preventing the spread to distant organs. Of note, the tumour in the breast and axillary involvement may respond differently. In recent years, indications for neoadjuvant therapy also have been extended to non-locally-advanced breast cancers, such as small (less than 2 cm in diameter) triple-negative cancers (those which are oestrogen-receptor, progesterone receptor, and human epidermal growth factor receptor 2 negative) [62].

Breast US has been shown to be a useful tool for early prediction of pathologic response to this kind of treatment [63] and may potentially aid the optimisation of therapy in case the of poor response, allowing for a prompt regimen change. However, it has to be noted that in this setting breast MRI has been shown to offer the best performance [64–67].

### Patients with breast implants

Breast US is usually the first line examination performed in women with implants to investigate breast implant complications that may present with pain, lumps, or asymmetries. It can be used to detect alterations of the implant structure, typically subdivided into intracapsular ruptures (when the implant envelope is broken but the silicon remains inside the capsule) and extracapsular ruptures (when the silicone leaks out of the broken capsule) [68]. Of note, the fibrotic *capsule* around the implant develops through a natural foreign body reaction of the breast tissues to the implant.

Considering breast implant integrity, US is a very specific, although not very sensitive, method: if an implant rupture is suspected on US, the probability of a true rupture is high; conversely, if no rupture is visible on US, a rupture (mostly intra-capsular) is still possible. In addition, US is useful in diagnosing other implant complication such as infection, seroma, or granuloma. MRI is the usual second step after US in this setting, especially for detecting intracapsular ruptures unnoticed with US [33, 69].

There are no contraindications to performing US-guided interventions (biopsy, preoperative localisation) in women with breast implants [17].

- ***Note F.*** *If you have breast implants, for either aesthetic reasons or from oncoplastic surgery, and a breast examination is planned (including US), please give the radiologist a complete information about the type of your breast implant, if this information is available to you. There are many implant types (e.g.,* *double or single lumen, silicone-only or silicone and saline solution, etc.)* *and the diagnostic performance is increased when the radiologist is aware of the type of implant you have.*

### Intraoperative US lesion identification and US of specimens

Although it is not commonly used in clinical practice, some experiences have been reported about the use of US for perioperative lesion identification in breast conserving surgery, with the aim of better margin management [70, 71]. Ultrasound of the surgical specimen may be used for evaluating the presence of the lesion and resection margin status [71–74], especially in the case of non-palpable lesions not associated with calcifications or not identifiable at mammography.

## The breast US report

Breast US should be performed by a dedicated breast radiologist who is also skilled in mammography and image-guided interventions. The report should start with *a premise containing relevant clinical information and the indication to the examination*. A short statement on the *technique* can be useful in documenting the use of a state-of-the-art equipment. In the descriptive section, the *findings* should be detailed, including abnormalities in the breasts and, when explored, the axillae. The descriptive section should include: laterality, the location of the finding(s) using the subdivision into four quadrants (upper external, lower external, lower internal, upper internal) plus the retro-areolar region and/or the clockwise position and distance from the nipple; the sonographic features; the size using the maximal diameter; the possible association with findings at clinical examination or on other imaging modalities. Concordance or difference in comparisons with previous breast US or other imaging examinations must be reported. The report should end with a concise *conclusion*, including a stardardised assessment category and management recommendations. Relevant verbal discussions between the interpreting physician and the referring clinician or the patient can be documented (e.g., in the original report or in an addendum to the report).

In most countries, a structured reporting and classification system is adopted for describing breast US findings and guiding management. In some European countries, especially in the United Kingdom, a five-level scale from U1 to U5 is used, where U stands for US. U1 means *normal (no abnormalities)*, U2 *benign abnormal findings*, U3 *indeterminate (equivocal)/probably benign findings*, U4 *findings suspicious of malignancy*, U5, *findings highly suspicious of malignancy* [75]. The most commonly applied system is the ACR *Breast Imaging Reporting And Data System* (BI-RADS) [21]. BI-RADS 1 means *normal (no abnormalities)*, BI-RADS 2 *benign abnormal findings*, BI-RADS 3 *probably benign findings* (for which a short-interval – typically 6-month – follow-up is recommended), BI-RADS 4 *suspicious findings* for which needle biopsy is recommended, BI-RADS 5 *findings highly suggestive for malignancy* for which needle biopsy is recommended, BI-RADS 6 *known biopsy-proven malignancy* (reserved for US examinations performed made for cancer staging or monitoring of neo-adjuvant therapy). Finally, this system also includes a BI-RADS 0 category, which is used for *incomplete/inconclusive tests requiring additional imaging evaluation*. Note that both the U4/BI-RADS 4 and U5/BI-RADS 5 diagnostic categories require tissue sampling through biopsy. In contrast to the U3 category (which includes cases with a relatively higher probability of cancer), the BI-RADS 3 category implies a low cancer risk (lower than 2%) and requires short term (usually 6 months) follow-up but may also be biopsied if the patient requests it or when follow-up is deemed difficult [21].

- ***Note G.*** *Even though the use of stardardised assessment categories facilitates the understanding of the report, do not hesitate to discuss unclear issues of your US examination with your breast radiologist. Do this in particular in the case of U3 or BI-RADS 3, if you are wondering about the need to undergo a needle biopsy or you are thinking of postponing the needle biopsy, as well as in the case of U4/BI-RADS 4 or U5/BI-RADS 5 and when further imaging work-up is suggested (e.g., digital breast tomosynthesis, MRI).*

## Conclusions

Ultrasound is a safe and widely available method for breast imaging. It is the method of choice when assessing young women (under age 40) with a palpable lump and a complementary method after mammography in older women with a palpable lump. Breast cancer screening with breast US alone is discouraged, while supplemental screening with US may be an option to be considered for women with dense breasts, even though associated with a high false positive rate. When a tissue diagnosis is needed for a suspicious lesion, US-guided biopsy should be preferred if the lesion is clearly identified with this method.

## Frequently asked questions (FAQs)

1. **Is breast US a harmless investigation?**

*Yes. To the best of our knowledge, there are no reported harms related to the delivery of US to the adult human body at the level of energy applied in diagnostic medical use* [4, 76]*.*

2. **Can breast US be performed instead of mammography for breast cancer screening in the general female population, as it spares radiation?**

*No. US has not been shown to reduce mortality from breast cancer when used a stand-alone approach in the general female population, and it causes far more biopsies and short-term follow-up examinations than mammography.*

3. **After a breast US examination, does the woman also need mammography?**

*The answer depends on the setting. As mentioned above, US is not accurate enough to be considered a stand-alone screening method in the general female population. It can be considered as an adjunct to mammography in asymptomatic women for certain specific categories (e.g., women with dense breasts) and may be the first-choice, however often not the only method needed, to correctly assess a woman with palpable lump, regardless of age, breast density, or breast cancer risk. Ask your radiologist to find out if you need a mammogram after a US or a US after a mammogram. She/he will be able to answer the question and to explain the reasons for yes or no.*

4. **Can women consider breast US being free from any potential negative consequences?**

*No. If breast US is not indicated and is performed, for example for screening in very young women at average risk for breast cancer, there is a non-negligible chance of false positive findings which could require unnecessary further work-up including needle biopsy or additional imaging procedures, causing anxiety to the woman and costs to the health care system. In addition, women should not forget that if US is used as a stand-alone screening tool instead of mammography, cancers could be missed, especially in fatty breasts.*

5. **After receiving the result of a breast handheld US, can women try to get a reliable second opinion on the test?**

*This possibility is limited by important technical reasons. If the original examiner missed a lesion, no documentation exists of this. Thus, in the vast majority of cases, the only way to get a real second opinion is for the breast US examination to be repeated by another examiner. This underlines the importance of selecting quality-assured/certified breast imaging centres for the original breast US examination. Notably, this is not true for mammography, which can be read by a second radiologist without it being necessary to repeat the test, (with only few exceptions due to technical deficits of the original mammogram).*
